# Self-Medication Practices and Associated Factors in the Prevention and/or Treatment of COVID-19 Virus: A Population-Based Survey in Nigeria

**DOI:** 10.3389/fpubh.2021.606801

**Published:** 2021-06-04

**Authors:** Anthony Ike Wegbom, Clement Kevin Edet, Olatunde Raimi, Adeniyi Francis Fagbamigbe, Victor Alangibi Kiri

**Affiliations:** ^1^Department of Community Medicine, College of Medical Sciences, Rivers State University, Port Harcourt, Nigeria; ^2^Department of Planning, Research and Statistics, Rivers State Primary Health Care Management Board, Port Harcourt, Nigeria; ^3^The Challenge Initiative (TCI), Port Harcourt, Nigeria; ^4^Department of Epidemiology and Medical Statistics, Faculty of Public Health, College of Medicine, University of Ibadan, Ibadan, Nigeria; ^5^Health Data Science Group, Division of Population and Behavioral Sciences, School of Medicine, University of St Andrews, St Andrews, United Kingdom; ^6^Department of Mathematics, Physics and Electrical Engineering, Northumbria University, Newcastle upon Tyne, United Kingdom; ^7^Department of Pharmacy, Faculty of Pharmaceutical Sciences, University of Port Harcourt, Port Harcourt, Nigeria

**Keywords:** knowledge level, self-medication, COVID-19, determinants, Nigeria

## Abstract

**Background:** The anxiety caused by the emergence of the novel coronavirus disease (COVID-19) globally has made many Nigerians resort to self-medication for purported protection against the disease, amid fear of contracting it from health workers and hospital environments. Therefore, this study aimed to estimate the knowledge level, causes, prevalence, and determinants of self-medication practices for the prevention and/or treatment of COVID-19 in Nigeria.

**Methods:** A web-based cross-sectional survey was conducted between June and July 2020 among the Nigerian population, using a self-reported questionnaire. Statistical analysis of descriptive, bivariate, and multivariate analyses was done using STATA 15.

**Results:** A total of 461 respondents participated in the survey. Almost all the respondents had sufficient knowledge about self-medication (96.7%). The overall prevalence of self-medication for the prevention and treatment of COVID-19 was 41%. The contributing factors were fear of stigmatization or discrimination (79.5%), fear of being quarantine (77.3%), and fear of infection or contact with a suspected person (76.3%). The proximal reasons for self-medication were emergency illness (49.1%), delays in receiving hospital services (28.1%), distance to the health facility (23%), and proximity of the pharmacy (21%). The most commonly used drugs for self-medication were vitamin C and multivitamin (51.8%) and antimalarials (24.9%). These drugs were bought mainly from pharmacies (73.9%). From the multivariable logistic regression model, males (OR: 0.79; 95% CI: 0.07–0.54), and sufficient knowledge on SM (OR: 0.64; 95% CI: 0.19–0.77) were significantly associated with self-medication.

**Conclusion:** The key finding of this study was the use of different over-the-counter medications for the prevention (mainly vitamin C and multivitamins) and treatment (antibiotics/antimicrobial) of perceived COVID-19 infection by Nigerians with mainly tertiary education. This is despite their high knowledge and risk associated with self-medication. We suggest that medication outlets, media and community should be engaged to support the rational use of medication.

## Introduction

The index case of novel coronavirus disease (COVID-19) was first described in Wuhan, China in December 2019 (1, 2). Since then the disease has been reported in more than 215 countries spread across the continents. About 120, 268, 427 confirmed cases and 2, 659, 802 deaths were reported globally as of March 18, 2021 (2). It was declared a global pandemic by the World Health Organization (WHO) on March 12, 2020 (3). COVAX COVID-19 vaccines are now available in Nigeria, though in limited quantities that are available to health care and frontline workers in phase 1. The pandemic is still a cause of concern because of inadequate doses and negative perception of the vaccine by the general population. In Nigeria, 161,737 confirmed cases of the disease and 2,030 deaths were reported by the Nigeria Center for Disease Control (NCDC) as of March 22, 2021 (4).

The disease created widespread anxiety and fear among the population in sub-Saharan Africa (5), principally because of the increase in confirmed cases in Africa as well as the high fatality in America and Europe (3), worsened by the fact that there is no approved vaccine or medication for its treatment. Consequent to this, many people, especially those feeling unwell have resorted to the consumption of different substances, including traditional medicine to treat a perceived COVID-19 infection or to prevent it, without considering the safety and efficacy of the substance to the human body (6). The consumption or use of these substances without expert advice from medical professionals is considered as self-medication (SM). Self-medication is defined as the consumption of medicines by individuals to treat self-recognized illnesses or symptoms without consulting a physician (7–9).

Many Nigerians have resorted to SM since the outbreak of COVID-19 in the country for purported protection against the disease as a result of the fear of contracting it from health workers and hospital environments, instead of accessing medical care from physicians at the health facilities. Thus, many deaths due to COVID-19 are linked to the practice of SM (10). Self-medication may also impact on the health of individuals negatively by way of toxicological and pharmacological risks associated with the improper use of medicines (10). Nigeria already had a high prevalence of SM before the COVID-19 pandemic, varying between 52.1 and 92.3% (11–16). Babatunde et al. found that 51% had ever practiced SM while 32% currently practiced SM among health workers in a tertiary institution in South-West Nigeria (12). Similarly, Ezeoke reported that two-thirds of undergraduate students in a Nigerian University practiced self-medication (15) while Oshikoya et al. found a similar rate of infants being treated for colic without medical advice in Lagos, south-west Nigeria (16). All these SM practices occur despite its documented contribution to the emergence and spread of antimicrobial drug resistance (12).

The sale of prescription drugs as over the counter (OTC) drugs and the operation of too many unregistered patent medicine stores/pharmacies without adequate check has been a challenge (14, 17), and so the results from any study that is aimed at evaluating self-medication practices and possible factors associated with the practice, may be useful to the relevant stakeholders and policymakers responsible for the management of COVID-19 patients.

Therefore, our study aimed to evaluate the awareness of SM in the context of COVID-19 in the Nigerian Population. An additional aim was to estimate the prevalence of SM and its determinants in the population.

## Methods

### Study Design and Participants

A web-based cross-sectional survey was conducted between June and July 2020, using electronic platforms such as WhatsApp and Facebook for access to the questionnaire by interested respondents. We opted for web-based survey over physical questionnaire administration following the existing COVID-19 preventive regulations as of the time of data collection. In Nigeria, COVID-19 prevention guidelines of physical distancing were advocated and enforced by the NCDC and WHO. The minimum sample size of 384 was calculated using the Cochran formula for cross-sectional studies at 95% confidence level and 5% error margin, and based on an estimated SM prevalence of 52.1% from a previous study (12). A total of 461 respondents had participated in the study at 11.59 pm on July 30, which we considered as adequate for a large population (18). The inclusion criterion was that a participant must be resident in Nigeria. Been a web-based data collection study, participants is therefore limited to those who are literate, have access to smart phone and internet.

### Questionnaire Design

The questionnaire was divided into two sections- demographics section, and another section containing questions on knowledge, causes, and practice (KCP) of SM. The demographic variables included age, gender, marital status, religion, education level, occupation, and average income per month. The knowledge questions had three items (K1-K3), while the causes and practice of SM questions contained seven (C1-C7) and eleven (P1-P11) items, respectively. A sample of the questionnaire is available in the Supplementary Material.

### Data Collection

Data were collected using questionnaires administered electronically between June 1, 2020 and July 30, 2020. The questionnaire was developed and validated by the authors, based on a previous study (19). Though, Cronbach's Alpha (CA) was calculated as 0.76, suggesting better validity and reliability of the questionnaire. Due to COVID-19 safety guidelines, the study utilizes convenience sampling method and authors used their contacts and media platform to reach the respondents. Through these contacts questionnaire was shared among the populations that have access to smart phones in cities and village where there was availability of internet.

### Statistical Analysis

Data were downloaded from the google forms using the **c**omma-**s**eparated **v**alues (CSV) option and exported into STATA version 15 (Stata Corp, College Station, TX, USA) which was used for the statistical analysis. For the purpose of analysis, the correct answer was scored as one, and any other as zero. Thereafter, the total score was converted to a percentage to create the two categories: insufficient knowledge (<49%) and sufficient knowledge (≥50%) (11). The baseline characteristics of the study participants were analyzed using descriptive statistics. The frequencies and percentages were used to present the categorical variables; mean and standard deviation were used to present continuous variables.

The associations between the practice of SM and the demographic variables as the explanatory variables were tested using the Chi-square test. The binary logistic regression model was used to determine the factors associated with SM practice. The response was binary: practiced SM vs. did not practice SM for COVID prevention. We estimated the crude from bivariate models and adjusted odds ratios (ORs) from multivariable models, together with their 95% confidence intervals (CI) and *P*-values. *P*-value < 0.05 and 95% CI, not including unity, were considered statistically significant.

## Results

### Description of Study Participants and Their Prevalence

A total of 461 respondents participated in the survey with a mean age of 42.2 years with a standard deviation of 10.7 years. Among the participants, 57.1% were female, 71.7% were married, 88.7% were employed, 87.8% had attained tertiary education, 95.8% belonged to the Christian religion, and 80.9% had monthly incomes >50,000 Nigerian Naira ($130.00). The proportion of respondents that had sufficient knowledge about SM was 96.7%. The overall prevalence of SM for perceived treatment or prevention of COVID-19 was 41% and among respondents aged <24 years, with below tertiary education, of Islamic religion and who had insufficient knowledge of the risks of SM, the prevalence figures were notably higher than 30%. These are summarized in Table 1.

**Table 1:** Characteristics of study participants and prevalence of self-medication.

**Variable**	**Frequency**	**Percentage**	**Prevalence of self-medication**	**χ2 *P*-value**
**Gender**				
Female	263	57.1	22.4	0.038^*^
Male	198	42.9	20.2	
**Age as at last birthday (years)**				
<24	25	5.4	36.0	0.258
25–34	78	16.9	20.5	
35–44	157	34.1	21.0	
45–54	154	33.4	19.5	
>55	47	10.2	23.4	
Mean ± SD (years)	42.2 ± 10.7			
**Marital status**				
Married	327	71.7	21.4	0.862
Not married	129	28.3	20.1	
**Occupation**				
Employed	402	88.7	20.2	0.547
Unemployed	51	11.3	21.6	
**Educational level**				
Below tertiary	56	12.2	30.6	0.017^*^
Tertiary	405	87.8	20.3	
**Religion**				
Christianity	439	95.8	20.7	0.834
Islam	19	4.2	31.6	
**Monthly income (NGN)**				
<10,000	24	5.4	16.7	0.543
10,000–50,000	61	13.7	23.0	
>50,000	361	80.9	19.7	
**Knowledge on self-medication**				
Insufficient knowledge	15	3.3	60.00	0.021^*^
Sufficient knowledge	446	96.7	20.2	
Total	461	100.0	41.0	

* *Significant at 5% level of error. 1 USD = 386.84 NGN*.

### Causes for Self-Medication for COVID-19

**Figure 1:**
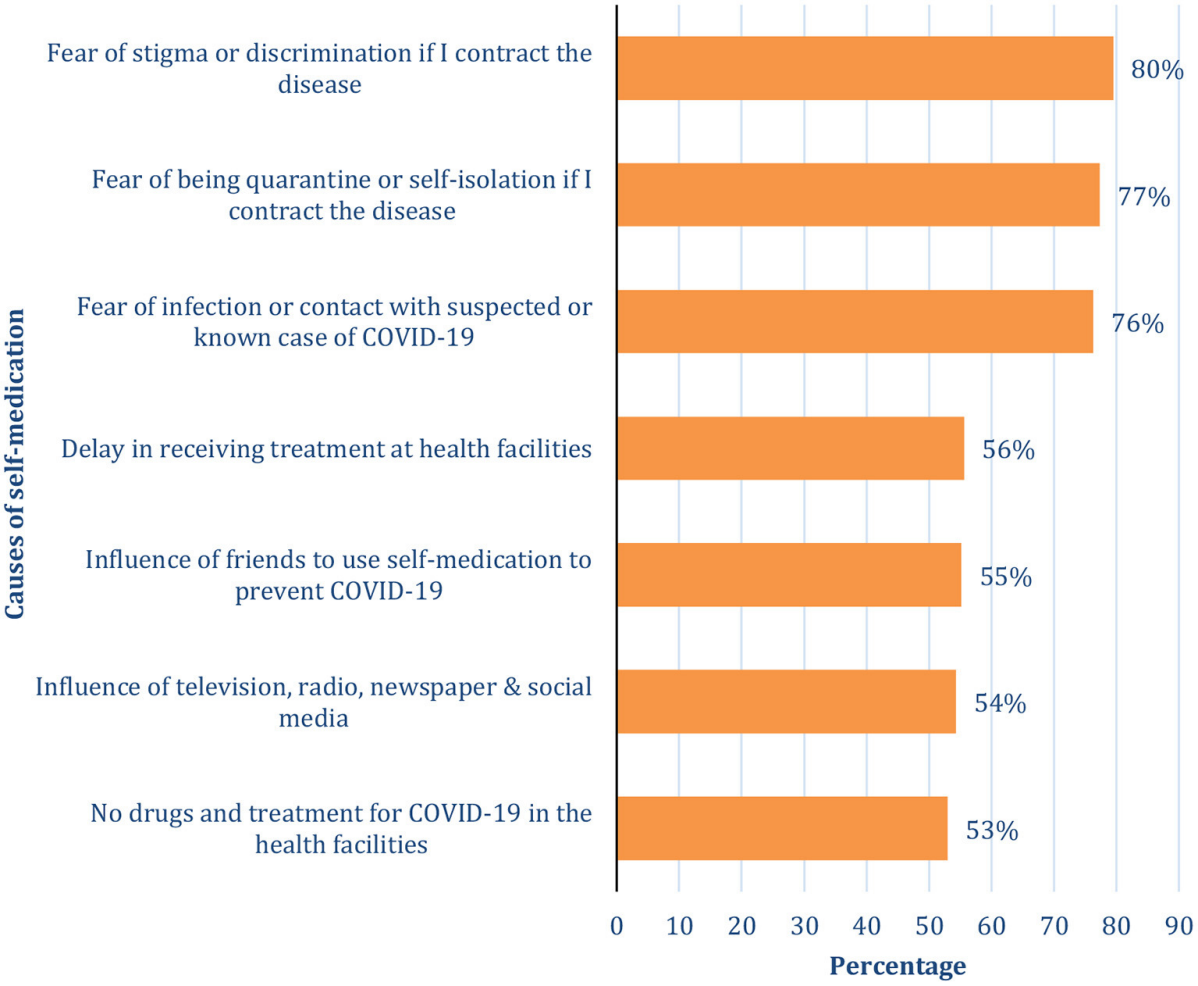
Causes of self-medication among the respondents.

Respondents were asked multiple-response questions on why they practiced SM. Figure 1 shows the list of reasons that were given. As shown in Figure 1, SM for COVID-19 prevention and/or perceived treatment was mostly caused by fear of stigmatization or discrimination (79.5%), fear of quarantine or self-isolation (77.3%), and fear of infection or contact with a suspected or known COVID-19 case (76.3%). Other reported reasons were “delay in receiving treatment at health facilities” (55.6%), “influence of friends to use self-medication to prevent or treat COVID-19” (55.2%), “television, radio, newspaper, and social media can influence self-medication for COVID-19” (54.3%) and “non-availability of drugs for COVID-19 treatment in the health facilities” (53%).

### Self-Medication Practices for the Treatment and/or Prevention of COVID-19

Table 2 shows the SM, the reasons for SM, and the status of the person that recommended the medication. Most of the respondents gave emergency illness (49.1%) as the reason for SM, other reasons given were delay in getting hospital services (28.1%), distance to the health facility (23%), the proximity of the pharmacy (21%), non-availability of medicine in a health facility (19.3%) and Health facility charges (15.3%). Table 2 also revealed that more than half of the respondents prescribed the medication themselves (53.7%), 35.4% got the prescription from medical personnel in a health facility, 24.8% got theirs from the workers in pharmacy, and 16.0% from their friends.

**Table 2:** Self-medication practices.

**Questions**	**Frequency**	**Percentage**
1) Taking drugs intended to prevent/treat COVID-19 without prescription by medically qualified personnel? (*n* = 461)	189	41.0
2) ^*^Reasons for taking medication without prescription by medically qualified personnel was due to: (*n* = 461)		
Emergency illness	173	49.1
Distance to the health facility	81	23.0
Proximity of the pharmacy	74	21.0
Health facility charges	54	15.3
No medicine in health facility	68	19.3
Delaying of the hospital services	99	28.1
Others	14	4.0
3) ^*^Prescription of the medication was by: (*n* = 461)		
Medical personnel from health facility	104	35.4
Worker in the pharmacy	73	24.8
Friend	47	16.0
Myself	158	53.7

* *Multiple responses*.

**Figure 2:**
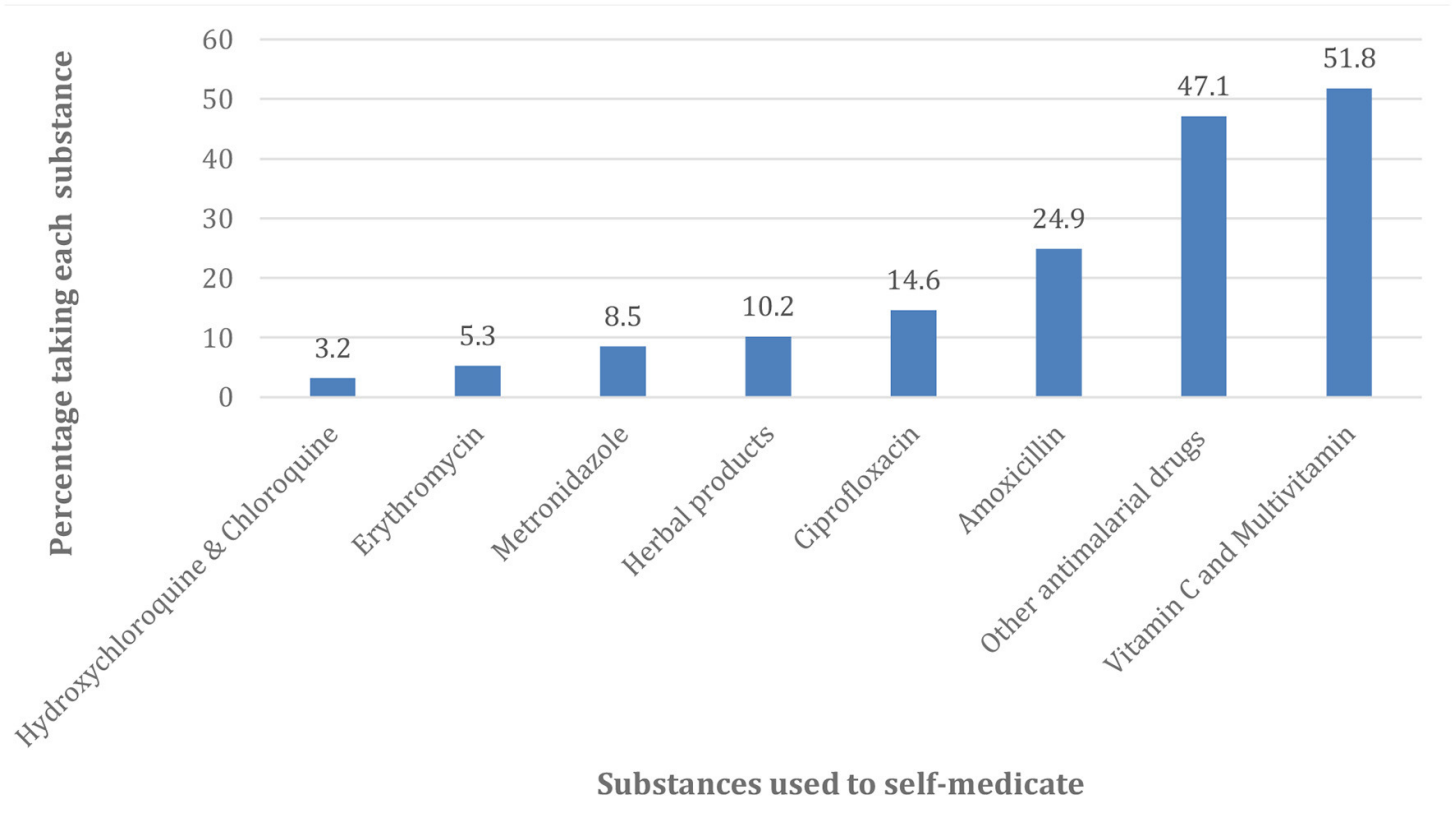
Distribution of Substances used for self-medication.

**Figure 3:**
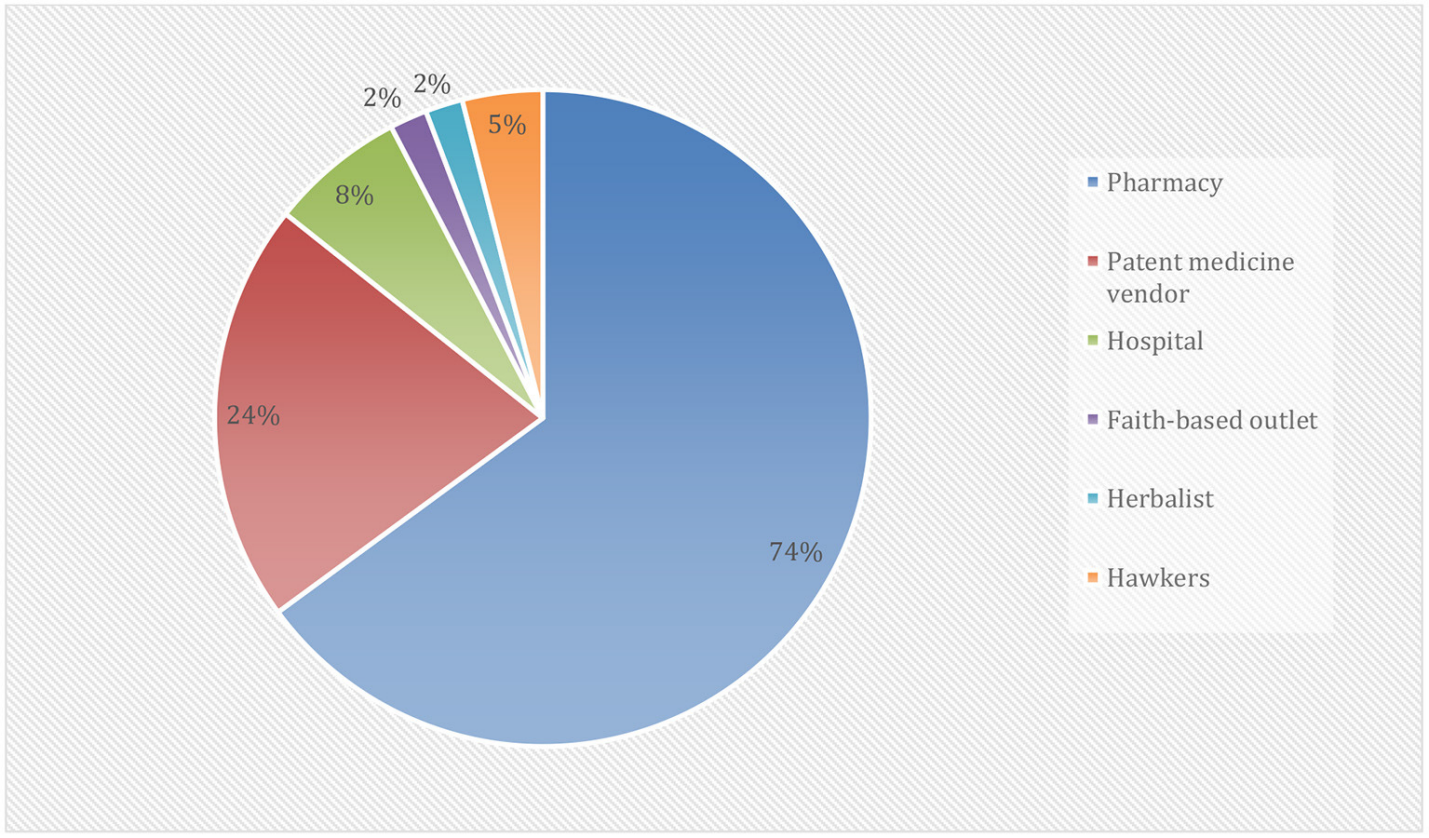
Source of medicine used for self-medication.

Figure 2 revealed that most of the drugs used for self-medication in the treatment and prevention of COVID-19 were Vitamin C and Multivitamin (51.8%) and antimalarial drugs other than Hydroxychloroquine and Chloroquine (47.1%). Others were Amoxicillin (24.9%), Ciprofloxacin (14.6%), Herbal products (10.2%), Metronidazole (8.5%), Erythromycin (5.3%), and Hydroxychloroquine and Chloroquine (3.2%). Figure 3 showed that the majority of the respondents bought their drugs for self-medication at the pharmacy (73.9%). Other places of the purchase were patent medicine vendor (23.6%), hospital (7.6%), hawkers (4.5%). Those who bought the medication at faith-based outlets and herbalists were of the same proportion (2.1%).

### Factors Associated With Self-Medication Practices for Perceived COVID-19 Treatment or Prevention

In the bivariate logistic regression model, gender, educational level, and knowledge about SM were significantly associated with the practice of SM in the prevention and/or perceived treatment of COVID-19 at *p*-value <0.05. The males were 14% [odds ratio (OR): 0.86; 95% Confidence Interval (CI): 0.03–0.41] less likely to indulge in SM than the females. Those who had tertiary education were more (OR: 1.60; 95% CI: 1.08–2.29) likely to self-medicate than those with lower level of education. Furthermore, those who had sufficient knowledge on SM were less likely to practice SM compared when compared with those with insufficient knowledge (OR: 0.79; 95% CI: 0.16–0.59) as shown in Table 3. Whereas, age, marital status, occupation, religion, and income were not statistically significant. On the other hand, after controlling for other variables in the multivariable logistic regression model, the odds of SM practice were generally lower among males (OR: 0.79; 95% CI: 0.07–0.54) compared with the females and among those with sufficient knowledge of SM (OR: 0.64; 95% CI: 0.19–0.77) than those that have insufficient knowledge about SM.

**Table 3:** Crude and adjusted factors associated with self-medication.

**Variable**	**Crude estimate**		**Adjusted estimate**	
**Overall**	**COR (95% CI)**	***P*-value**	**AOR (95% CI)**	***P*-value**
**Gender**				
Female	1.00		1.00	
Male	0.86 (0.03–0.41)	0.038^*^	0.79(0.07–0.54)	0.023^*^
**Age as at last birthday**				
<24	1.00		1.00	
25–34	2.69(0.85–8.48)	0.091	2.10(0.50–8.89)	0.311
35–44	1.23(0.45–3.37)	0.689	0.93(0.23–3.77)	0.916
45–54	1.42(0.51–3.92)	0.497	0.98(0.24–4.07)	0.988
>55	2.01(0.53–7.53)	0.638	1.45(0.27–7.66)	0.663
**Marital status**				
Married	1.00		1.00	
Not married	1.05(0.60–1.81)	0.862	0.97(0.51–1.83)	0.924
**Occupation**				
Employed	1.00		1.00	
Unemployed	1.30(0.55–3.08)	0.553	1.81(0.48–6.72)	0.376
**Educational level**				
Below tertiary	1.00		1.00	
Tertiary	1.60(1.08–2.29)	0.017^*^	1.71(0.30–1.69)	0.443
**Religion**				
Christianity	1.00		1.00	
Islam	0.89(0.31–2.53)	0.833	0.76(0.23–2.49)	0.656
**Monthly income (NGN)**				
<10,000	1.00		1.00	
10,000–50,000	0.56(0.16–1.93)	0.360	0.84 (0.18–3.86)	0.825
>50,000	0.91(0.30–2.71)	0.864	1.63 (0.36–7.39)	0.523
**Knowledge on self-medication**				
Insufficient knowledge	1.00		1.00	
Sufficient knowledge	0.79 (0.16–0.59)	0.021^*^	0.64 (0.19–0.77)	0.042^*^

* *Significant at 5% level of error*.

## Discussion

This study aimed to estimate the knowledge, causes, prevalence, and determinants of self-medication practices for the prevention and/or treatment of COVID-19 in Nigeria. Though self-medication for minor illnesses is approved WHO, but with caution (7), our study investigated the use of self-medication for perceived COVID-19 prevention/or treatment. To the best of our knowledge, this study is novel in Nigeria as far as COVID-19 is concerned, although there are previous studies on self-medication practices other than COVID-19 both in Nigeria and elsewhere.

The estimation and quantification of the knowledge level, prevalence, and the reasons for self-medication practices for COVID-19 perceived treatment or prevention, as well as associated determinants, are of great importance due to their consequent effect on the fight to control and mitigate the disease. Our study has demonstrated sufficient knowledge for self-medication among respondents, namely as high as 96.7%. This finding is similar to previous studies in Nigeria (11, 12) and overseas (8, 19), where a sizeable proportion of the respondents had sufficient knowledge of self-medication. From our study, the prevalence of self-medication for COVID-19 treatment or prevention was 41%. This finding was comparably higher than what was earlier reported in Togo (20). We also observed a prevalence of above 30% among respondents <24 years, that attained below tertiary education, of Islamic faith, and with insufficient knowledge of self-medication. Our findings were similar to previous studies on self-medication (21–24). The reason for the high prevalence was attributed to the ease of access to OTC drugs, the presence of unregistered medicine stores, and pharmacies (14, 15).

Our study identified the reasons for self-medication for perceived treatment or prevention of COVID-19 in Nigeria, as fear of stigmatization or discrimination, fear of being quarantined or self-isolation, fear of infection, or contact with a suspected or known case of COVID-19. The list of reasons also included a delay in receiving treatment at the health facilities, influence of friends, unavailability of drugs for the treatment, and influence of media. The others were emergency illness, delay in receiving hospital services, distance to the health facility, and proximity of the pharmacy. We acknowledge the fact that some of our findings are different from those reported previously by earlier studies for self-medication, although these were not for COVID-19. Nevertheless, most of our findings have also been previously reported elsewhere. For instance, emergency illness had been reported as a reason for self-medication (19, 25); others were delay in receiving treatment at health facilities and hospital services (19, 20, 26), the influence of friends (25, 27), unavailability of drugs, distance to the health facility, proximity of the pharmacy to home and charges at a health facility (19, 25), as well as stigmatization (20) and influence of the media (20, 21).

Our findings indicated that out of those who self-medicated for the perceived treatment or prevention of COVID-19, more than half of the participants had prescribed the drugs by themselves, over one-third got their prescriptions from medical personnel and some others got theirs either directly from the pharmacies or through recommendation by friends. These findings were in line with studies on self-medication in Nigeria (17), Peru (22), Kenya (23), Dhaka (24), Pakistani (26), Eritrea (9), Iran (28), and Saudi Arabia (29). As with other studies (12, 23, 30), ours showed that of all those who self-medicated in the last 3 months, more than half, only self-medicated once, while others indulged in self-medication at least once a week. As our results suggested, the possible reason for this huge difference between those who self-medicated once and those who did so at least once a week, might be attributed to either side effects, or the relief from symptoms of the disease.

The most common drugs used for self-medication in the perceived treatment or prevention of COVID-19 were Vitamin C and Multivitamins, as well as antimalaria drugs other than Hydroxychloroquine/Chloroquine. Others were Amoxicillin, Ciprofloxacin, Herbal products, Erythromycin, Metronidazole, and Hydroxychloroquine and Chloroquine. The possible explanation for the high usage of Vitamin C and Multivitamin by the participants might be because Vitamin C had been reported to have significant potency and efficacy in the management of COVID-19 (31), as well as the availability of these products without restriction and control. Furthermore, the claim of a possible association between COVID-19 and Malaria might be responsible for the high consumption of antimalaria drugs among the study participants.

The disaggregation of Hydroxychloroquine and Chloroquine from other antimalaria drugs was informed by the non-randomized trial conducted in French which seemed to suggest a positive outcome in the treatment of COVID-19 patients, particularly those that received Hydroxychloroquine (600 mg/day) (32), and the claim by the American President, Donald Trump that he used Hydroxychloroquine to prevent COVID-19 infection (33). Like other self-medication studies (14, 19–21), our study showed that some of the participants also used antibiotics, such as Amoxicillin, Ciprofloxacin, Erythromycin, and Metronidazole either as a perceived treatment or prevention of COVID-19. In our study, 10.2% of the participants used herbal products for similar purposes. This could be explained by the fact that traditional medicines were frequently used in Nigeria for the treatment of diseases (34, 35), and also because of the availability and low cost of herbal products in African countries (20). It is interestingly worthy of note, that the WHO has welcomed innovations around the world, including traditional medicines/herbal products in the search for potential treatments for COVID-19 (6).

Pharmacy and patent medicine vendors were identified as a significant source of drugs and substances used for self-medication among our study participants. Our results agreed with those reported by other studies in Nigeria and across the globe (9, 12, 14, 23, 30). The explanations might include the fact that most of the study participants were of high socioeconomic status in terms of employment, educational attainment, and monthly income. Another prominent reason for high pharmacy patronage of self-medication drugs was the inability of government or relevant authorities to regulate and control the pharmacy and patent medicine stores, such that medications were and are still being dispensed and purchased at these stores without a check (14, 15).

Self-medication for COVID-19 prevention and/or perceived treatment was significantly associated with gender, educational attainment, and knowledge level on SM in our study. The odds of SM among those who had sufficient knowledge were 64% lesser compared with those with insufficient knowledge. A likely possibility would be that the knowledgeable members might be more fearful of the bad adverse reactions associated with self-medication (25). Our study also indicated that the odds of SM for the COVID-19 were significantly lower among males by 79% than among females. This result was in agreement with those reported by some other studies on self-medication practices (36, 37), but in disagreement with the findings of some other studies (9, 25).

### Study Limitations and Strength

Our findings should be interpreted with caution as the findings are not generalizable on Nigeria population. We had used a web-based survey which restricted the respondents to mostly those that had education and the middle and upper economic class who could afford an internet-enabled phone from which data was collected. Therefore, caution must be exercised while interpreting our findings as it may not be generalizable on all residents in Nigeria. A study that will reach out to all segments of the population should therefore be carried out in Nigeria. However, this is probably the first study to explore the knowledge, causes, prevalence, and factors possibly associated with self-medication for perceived COVID-19 prevention and/or treatment among the Nigerian population. Another strength of the study lies in the fact that it showed that various medications were used by participants in the prevention (mainly vitamin C and multivitamins) and treatment (antibiotics/antimicrobials) of COVID-19 among the well-educated Nigerian population.

## Conclusion

The important finding of the study was the use of different over-the-counter medications for the prevention and treatment of perceived COVID-19 by the Nigerian with mainly tertiary education. Vitamin C, multivitamins, antimalaria, and antibiotic drugs were the most used medications for the prevention and treatment for COVID-19 infection because of fear of stigmatization or discrimination, fear of being quarantined, fear of contact with an infected person, emergency illness, and delaying of the hospital services. We suggest that medication outlets, media and community should be engaged to support the rational use of medication for the prevention/or treatment of perceived COVID-19 infection.

## Data Availability Statement

The raw data supporting the conclusions of this article will be made available by the authors, without undue reservation.

## Ethics Statement

The studies involving human participants were reviewed and approved by The Rivers State Health Research Ethics Committee approved the study (Number: RSHMB/RSHREC/11.20/VOL.8/063). Written informed consent to participate in this study was provided by the participants' legal guardian/next of kin.

## Author Contributions

AW and CE contributed to conception and study design. All authors performed the statistical analysis and interpretation of results and the drafting of the manuscripts. AF and VK reviewed the statistical analysis and revised the manuscript draft. All authors read and approved the final manuscript.

## Conflict of Interest

The authors declare that the research was conducted in the absence of any commercial or financial relationships that could be construed as a potential conflict of interest. The reviewer TF declared a shared affiliation with the author AF to the handling editor at time of review.
