# Supplementary material for: Self-Medication Practices and Associated Factors in the Prevention and/or Treatment of COVID-19 Virus: A Population-Based Survey in Nigeria
Source: Front Public Health. 2021 Jun 4;9:606801. doi: 10.3389/fpubh.2021.606801 (PMC8213209; doi:10.3389/fpubh.2021.606801)
Supplement: Supplementary file 1 [file Table_1.DOCX]

**Questionnaire on the knowledge and practice of self-medication for COVID-19 in Nigeria**

This questionnaire aims to collect information on the knowledge and practice of self-medication for COVID-19 by the population. This will provide evidence as to why people resort to self-medication, instead of seeking counseling and treatment at the health facilities. The findings will guide action to reduce self-medication for COVID-19 among the population.

Kindly fill this form noting that your responses will be treated with utmost confidentiality and will only be used for this study.

The burden for completing this form is estimated at 5 minutes.

Thank you.

|  | **You can tick the option whichever is applicable** | |
| --- | --- | --- |
|  | **Demographic characteristics** | **Options** |
| D1 | Age as at last birthday |  |
| D2 | Gender | Male / female |
| D3 | Marital Status | Single / Married / Separated / Divorcee / Widow / Cohabiting |
| D4 | Religion | Christianity / Islamic / Others |
| D5 | Educational level | Below tertiary/Tertiary |
| D6 | Occupation | Public servants (appointees) / Civil servant / Health care workers / Retirees / Self-employed / Unemployed / Students |
| D7 | Income (Naira) per year | <10,000 per month / between 10,000 and 50,000 / > 50,000 |
|  | **Knowledge on Self-medication** |  |
| K1 | Have you ever heard about self-medication? | Yes / No / I don’t know |
| K2 | Can self-medication practices result into harmful effect? | Yes / No / I don’t know |
| K3 | Is self-medication for COVID-19 better than seeking medical consultation? | Yes / No / I don’t know |
|  | **Causes of self-medication** | |
| C1 | Fear of infection or contact with suspected or known case of COVID-19 | Yes / No / I don’t know |
| C2 | Fear of being quarantine or self-isolation if I contract the disease | Yes / No / I don’t know |
| C3 | Fear of stigma or discrimination if I contract the disease | Yes / No / I don’t know |
| C4 | No drugs and treatment for COVID-19 in the health facilities | Yes / No / I don’t know |
| C5 | Delay in receiving treatment at health facilities | Yes / No / I don’t know |
| C6 | Influence of friends to use self-medication to prevent COVID-19 | Yes / No / I don’t know |
| C7 | Influence of television, radio, newspaper & social media can lead to self-medication for COVID-19 | Yes / No / I don’t know |
|  | **Self-medication practices** | |
| P1 | Did you self-medicate for COVID-19 in the last three months without prescription of medically qualified personnel? | Yes / No / I don’t know |
| P2 | Why did you use medication (s) without prescription instead of going to health facility? | Emergency illness / Distance to the health facility / Proximity of the pharmacy to home place / Health facility charges / No medicine in health facilities / Delaying of the hospital services / Others |
| P3 | What did you use for self-medication? (can be more than one) | Amoxicillin / Doxycycline / Tetracycline / Erythromycin / Chloramphenicol / Metronidazole / Ciprofloxacin / Antimalarial drugs / Herbal products / Others………………… |
| P4 | Who prescribed the medication (s) for you? | Medical personnel from health facility / Worker in the pharmacy / A friend / myself |
| P5 | Where did you buy the medication | Pharmacy / Patent medicine vendor / Hospital / Faith-based outlet / Herbalist / Hawkers / Others…………………. |
